# Childhood tuberculosis deskguide and monitoring: An intervention to improve case management in Pakistan

**DOI:** 10.1186/1472-6963-11-187

**Published:** 2011-08-10

**Authors:** Nauman Safdar, Sven Gudmund Hinderaker, Noor Ahmed Baloch, Donald A Enarson, Muhammad Amir Khan, Odd Morkve

**Affiliations:** 1 Association for Social Development, Islamabad, Pakistan; 2 Centre for International Health, University of Bergen, Norway; 3 National TB Control Programme, Federal Ministry of Health, Government of Pakistan; 4 International Union against Tuberculosis and Lung Disease, Paris, France

## Abstract

**Background:**

Childhood tuberculosis (TB) has been a neglected area in national TB control programme (NTCP) in high burden countries. The NTP Pakistan adapted the global approaches by developing and piloting its policy guideline on childhood TB in ten districts of the country. We developed an intervention package including a deskguide and a monitoring tool and tested with the ongoing childhood TB care in a district. The objective of our study was to measure effectiveness of intervention package with deskguide and monitoring tool by comparing TB case finding and treatment outcomes among districts in 2008, and performance assessment in intervention district.

**Method:**

An intervention study with cohort design within a routine TB control programme comparing case findings and treatment outcomes before and after the intervention, and in districts with and without intervention. We enrolled all children below 15 years registered at all nine public sector hospitals in three districts of Pakistan. The data was collected from hospital TB records.

**Results:**

In eight months during 2007 there were 164 childhood TB cases notified, and after intervention in 2008 a total of 194 cases were notified. In *intervention district *case finding doubled (110% increase) and correct treatment practice significantly increased in eight months. Successful outcomes were significantly higher in *intervention district *(37,100%) compared to *control district A *(18, 18%, p < 0.05) and *control district B *(41, 72%, p < 0.05).

**Conclusion:**

Childhood TB deskguide and structured monitoring was associated with improved case management and it augmented NTP policy. More development and implementation in all health services of the district are indicated.

## Background

Historically, childhood tuberculosis (TB) has remained a low priority for tuberculosis control programmes in low-income countries with high TB burden. The challenge of implementing an effective childhood TB control has been complicated by poor estimates of case load and inadequate quality of data reporting the cases [1]. Case management issues related to establishing accurate diagnosis [2], lack of resources and quality control have been additional constraints to childhood TB control. The World Health Organization (WHO), by recognizing its importance, has developed a special focus on childhood TB in the Stop TB Strategy [3]. There is an ongoing effort to get childhood TB into mainstream national TB control programmes (NTP) in resource limited settings. In the recent past, encouraging global efforts have been made, including the development of new programme guidelines by WHO [4], new recording and reporting tools with special sections on childhood TB [5] and initiatives on anti-TB drugs for children [6]. In addition support from donors such as the Global Fund for AIDS, Tuberculosis and Malaria is being provided to the national programmes and partners for the implementation of childhood TB control interventions.

To respond to the global public health recognition of childhood TB control, the NTP Pakistan adapted the global approaches by developing and piloting its policy on childhood TB [7] in 2006-2007 in the public sector hospitals in ten selected districts. The pilot included training of pediatricians on newly developed policy guidelines including recording and reporting the national TB forms, and supply of anti-TB drugs for children and tuberculin skin tests (TST). The NTP policy guidelines were comprehensive but they lacked operational components and simplicity, limiting its usefulness for health care providers. Based on the NTP Pakistan experience with adult TB control guidelines [8], an operational intervention package including a deskguide and a monitoring tool was developed and tested with ongoing childhood TB care in a district in year 2008 (eight months).

An intervention study on early implementation of this package has been conducted. The objective of the study was to measure the effectiveness of the intervention package by comparing the case finding and treatment outcomes among intervention and control districts in 2008, and comparing pre- and post-intervention performance in intervention district. Our hypothesis was that the implementation of the deskguide and monitoring would improve the case management (case finding and treatment outcomes) among children with tuberculosis.

## Method

### Study design

This was an intervention study imbedded within a routine TB control programme and was planned as a prospective cohort study comparing intervention and control districts, and also comparing before and after intervention. We included in the study all children aged less than 15 years who were diagnosed and registered as cases of TB at nine district and sub-district hospitals in three districts of the province of Punjab during the selected period. Children detected with TB at health facilities other than these hospitals were not included in the study. The study received ethical clearance from the National Bioethics Committee Pakistan.

### Intervention package

A childhood TB case management deskguide and structured monitoring was the intervention package. The childhood TB deskguide was developed on the concept of an adult TB deskguide [8] in Pakistan which since the year 2000 had been used to offer TB DOTS care in public and private sector health facilities. The case management technical and operational details were presented as short statements and decision tables for easy reference while being used as a desktop aid by the care givers. It has sections covering step by step the entire process of childhood TB care, i.e. from suspect screening to treatment completion. The title page of the deskguide contains the red flag signs related to child TB suspects that should prompt a care giver to consider using the deskguide. The different sections are organized in short chapters with descriptive headings including history and examination, investigations and interpretation, diagnosis and scoring chart, education, prescription, registration, treatment support and managing household contact, follow-up, retrieving delayed patients and treatment outcomes. An orientation session on the use of the deskguide was conducted in April 2008, initially for the pediatricians, clinicians and paramedics who were the front line care givers to the children in the district and sub-district level hospitals. The deskguide was followed by structured monitoring based on a check list having programme input and output variables and performance indicators developed for this purpose. The childhood TB monitoring was included as part of regular district led monitoring system by district TB coordinator (DTC). The DTC was oriented on the monitoring process and check list that he was supposed to apply while performing regular TB monitoring meetings within the district.

### Study sites and setting

The NTP selected ten districts in each of the four provinces of Pakistan to pilot childhood TB care. The selection of study districts was on evidence of functioning adult TB care in a district, geographic distribution and access, and willingness of districts to participate. We focused on province of Punjab for the study because of the availability of continued programme support, including anti-TB drugs for children, tuberculin skin test (TST) and monitoring arrangements, which made implementation feasible under programme conditions.

For the prospective study all three NTP pilot districts in Punjab where we had previously conducted our retrospective study were included [9]. These three districts represent southern, central and northern parts of the province. One district being centrally located was taken as an intervention district with the package of deskguide and structured monitoring for review of the early implementation experience which refers to the implementation of intervention where inputs and circumstances were kept close to routine programme arrangements for future replication. The remaining two districts were control districts A and B, with routine implementation of NTP childhood TB policy. Two controls were considered yielding additional strength for the study, whose statistical power was low, as it always is with pilots. The intervention district had a population of 2.9 million whereas the control districts had a population of 1.2 and 3.2 million respectively. The intervention and the control districts were comparable based on the socio-demographic profiles with a population density between 200 to 600 persons per sq. km, and an average household size of 7 persons [10]. In this population the estimated proportion of children below 2 years was 4.5%, of children 2-5 years it was 10.5% and in children 6-14 years was 26%. The literacy rate was estimated at 35% [11]. All childhood TB cases registered at these hospitals during the period under review were included in the study.

### Data collection and analysis

We compared results from 2007(pre-intervention) with 2008 (post-intervention), and we also compared the intervention with the two control districts A and B. All districts enrolled patients in the year 2007 when the policy on childhood TB was introduced by NTP. Then one district introduced the intervention 'desk guide' and 'structured monitoring' during 2008 (May-Dec). The comparison was between the same months of 2007 and of 2008 and for the latter we compared the intervention and the control districts.

The prime sources of data were the TB registers, TB treatment cards and quarterly reports. A researcher visited each hospital to review the patient records and extract relevant data, using a designated report form. The quality of data extraction was ensured by another researcher by cross-checking the extracted data for missing information and inconsistencies. Data were entered and analyzed using the SPSS version 15 software package. The comparison of the cohorts pre- and post-intervention related to notification, age groups, sex, type and treatment outcomes. For comparing group differences of categorical variables, Pearson Chi-square test was used. Student t-test was used for continuous variables. The level of significance was set at p < 0.05.

## Results

### Case notification

In a period of eight months during 2007 there were 164 childhood TB cases notified in the three selected districts, and after the intervention in 2008 a total of 194 cases were notified. Among them 117 were girls and 77 were boys (a ratio of 1.5). Table 1 compares the case notification trends in the intervention and the two control districts during the same months in the previous year. In the *intervention district *the case finding doubled (110% increase) in eight months, from 17 (0.6/100000) before to 37 (1.3/100000) after the implementation of the deskguide and structured monitoring. In the *control district A *the case finding was reduced by 6% (from 106 to 100) whereas in the *control district B *an increase of 30% (from 44 to 57) in case notification was observed during the same eight months.

**Table 1:** Case notification and treatment outcomes in children in intervention and control districts, comparing May-Dec 2007 and 2008

	*Intervention district* (n,%)				*Control district A* (n,%)				*Control district B* (n,%)			
	
	*(2007)		Intervention package *(2008)		*(2007)		*(2008)		*(2007)		*(2008)	
Childhood TB Cases	17		37		106		100		44		57	
Rates/100000 pop	0.6		1.3		9		8.6		1.4		1.8	
Outcomes												
*Treatment success*	13	(76%)	37	(100%)	1	(1%)	18	(18%)	29	(66%)	41	(72%)
*Failure*	0		0		0		0		0		0	
*Died*	0		0		0		0		0		0	
*Defaulted*	0		0		0		0		10	(23%)	8	(14%)
*Transferred*	1	(6%)	0		0		0		1	(2%)	0	
*Unknown*	3	(18%)	0		105	(99%)	82	(82%)	4	(9%)	8	(14%)
Total	17	(100%)	37	(100%)	106	(100%)	100	(100%)	44	(100%)	57	(100%)

* May-Dec 2007 and 2008

### Characteristics of TB in children

Table 2 compares the case notification among the districts by age groups and type of case (pulmonary or extra-pulmonary). During 2008 (eight months) a total of 1871 TB cases were notified (all age groups) among whom 194(10%) of the cases were children less than 15 years of age, and 53(27%) of the children were 0-5 years of age. A total of 130 pulmonary childhood TB cases were notified including 21(57% of all cases) in *intervention district *versus 76(76%) in *control district A *and 33(58%) in *control district B*. Among the pulmonary TB cases within the age group 0-5 years (41 cases, 32%) there were 3 cases who had their smear done, all negative. In age group 6-14 years (89 cases, 68%) there were 48 cases who had their smear done, 18 positive and 30 negative. In the intervention district all of the 21 pulmonary cases had their smear done and 6 (29%) were smear positive.

**Table 2:** Characteristics of TB in children: intervention and control districts (May-Dec 2008)

	Intervention		Control A		P-value	Control B		P-value
TB cases all ages	530		761		< 0.05	580		0.08
Childhood TB (< 15 y)	37	(7%)	100	(13%)		57	(10%)	
TB case notification rate/ 100000 population								
All ages	18		63			18		
Children < 15 y (95% CI)	1.3	(1.0-1.7)	8.6	(7.0-10.2)		1.8	(1.3-2.2)	
Age groups Age groups								
< 2 years	0		17	(17%)	< 0.05	3	(5%)	0.16
2-5 years	1	(3%)	27	(27%)		5	(9%)	
6-14 years	36	(97%)	55	(56%)		49	(86%)	
Total	37	(100%)	99	(100%)		57	(100%)	
Type of TB								
PTB	21	(57%)	76	(76%)	< 0.05	33	(58%)	
EPTB	16	(43%)	7	(7%)		24	(42%)	
Not specified	0		17	(17%)		0		
Total	37	(100%)	100	(100%)		57	(100%)	

Out of 17 cases notified in 2007 (8 months) and 37 cases notified in 2008 (8 months) in the intervention district, the prescription of a correct drug regimen was found among 13 (76%) in 2007 compared to 36(99%) cases in 2008.

### Treatment outcomes

The successful outcomes 'cured' plus 'treatment completed' were significantly higher in the *intervention district *(37 100%) as compared to the *control district A *(18 18%, p < 0.05) and *control district B *(41 72%, p < 0.05), as shown in the table 1. There was no case who defaulted from treatment in the *intervention *and *control district A *whereas 8 cases (14%) who defaulted in *control district B*. There was no unknown outcome in the *intervention district *whereas *control district A *had 82 (82%) and *control district B *had 8 (14%). The reported 'treatment success' by type of TB in the intervention district was 100% in 2008 compared to 88% before intervention in 2007.

## Discussion

Our study has shown a positive trend towards improvement in case notification and treatment outcomes associated with a childhood TB deskguide and structured monitoring, but no significant inference should be drawn from this early implementation of this package. The case notification varied substantially in the control districts during the study period and more synchronized case management efforts are required. We have previously shown that with introduction of the NTP policy alone there was an increase in case notification rate (1.4 to 5.2 per 100000) in three of the districts studied, with one district showing the major increase [9]. The deskguide being a case management tool provided an additional opportunity to serve as a reminder for clinicians. A study in China showed the usefulness of a deskguide in daily practice of county TB doctors [12]. The number of cases at baseline was lower in the intervention district compared to the controls, and this may partly account for the increased case finding, where starting low gives a high proportional increase. However, even in absolute numbers the increase was somewhat higher in the *intervention group *(20) compared to *control A *(13). The increase in number of cases being registered and notified does not necessarily mean that more children with TB being treated, as we have no parallel data on treatment in the non-reporting part of the private sector. The treatment outcomes showed a better improvement in the intervention than in the control district. The use of deskguide and structured monitoring is feasible within routine care after initial training, and can use the same staff, minimal additional resource allocation and monitoring within the same structure. However, the impact on TB control cannot be generalized from our early implementation experience. The results do not represent the total number of all childhood TB cases which may have occurred during these months in three districts studied, because the study focused on only public district level hospitals. To further increase case notification rates in these districts the NTP policy along with the intervention package may be implemented in all the health services of the district currently offering TB care, including the private practitioners. Standardizing the childhood TB case management protocol during scale-up process through a package of deskguide, structured monitoring and policy guide would require a) a set of structured training materials and b) participation of all who provide care to children including, pediatricians, clinicians and TB focal persons in district [13]. Improvement in case detection and case management and contact investigation are considered necessary components to control TB in children [14].

Only one case of TB aged below 5 years was notified in the intervention districts, and the desk guide had no impact on the age group 0-5 years as compared to the age group 6-14 years. This low case finding reflects the diagnostic challenge for the pediatrician in Pakistan. The age group below 5 years is considered as the group with highest risk when dealing with childhood TB, especially in high burden countries, with a higher age specific mortality rate than other age groups and high risk of severe forms of disease such as disseminated tuberculosis and tuberculous meningitis [15]. The deskguide guidelines had protocols to address the diagnostic issues related to infant and young children buts hands on training may be needed. Putting in place such techniques as gastric lavage in routine district level hospital settings requires more training and supervision and not the option at this point in time. The intervention district was adjacent to a metropolitan city with specialized TB hospitals diagnosing around 15000 TB cases each year representing all age groups (unpublished NTP reports). Such tertiary care hospitals have much better arrangements for diagnosing very young children with TB. It is possible that childhood TB suspects and cases would visit these well-known hospitals instead of attending the smaller hospitals in their own districts.

The recording and reporting of the notified cases improved after the deskguide and structured monitoring and was associated with a documented improvement in correct treatment practices. The improvement in case management practices is among the priorities in childhood TB control [16]. There is now a revised recording and reporting information system (TB R&R) which has sections on childhood TB. Children aged < 15 years should be registered separately for each category [5]. Children managed for TB should always be included in the routine TB R&R in order to get the number of cases identified, registered for treatment and with treatment outcomes [17].

Treatment outcomes seemed to improve with the desk guide and monitoring, but the study was based on early implementation experience, and inference cannot be drawn. Failure to report treatment outcome was identified as major implementation challenge during the period in which NTP policy was implemented resulting in unknown outcome in 14% and 82% in control districts and 0% in intervention district [9]. In Malawi and Papua New Guinea unknown outcome was reported in 21% and 23% of notified cases [18,19]. The global interventions such as revised TB R&R is among recent measures to promote high standard of individual patient care, adherence to treatment regimen and transfer of information between health facilities [5]. Our study supports the concept of operational research in which the research determines how such interventions are translated into benefit in the heterogeneous setting of routine care [20].

A strength of this prospective study is that it is based on review of available patient records under routine programme operations in collaboration with NTP and previous implementation experience of NTP childhood TB policy. A major limitation is that in this early implementation experience we cannot generalize the results, but it provided ideas for the process ahead. The private sector and the health facilities below sub-district level were not included. Furthermore the patients' records were often incomplete in the hospitals and it was not possible to follow-up patients by visiting their homes to get more information.

## Conclusion

The study has shown that implementation of an intervention package of childhood TB with a deskguide and structured monitoring was associated with improved case management and it augmented the NTP policy. It seemed to have more effect on the treatment outcome than on the case finding, and may have helped standardizing the process of care. There is a need to refine the intervention package involving the pediatrician, clinicians and paramedics in development and scale-up of childhood TB implementation in the country.

## Competing interests

The authors declare that they have no competing interests.

## Authors' contributions

NS has participated in study design, data entry and analysis, data interpretation, draft writing, editing and submission. SGH has participated in study design, data analysis, and data interpretation and editing whereas NAB, DAE, MAK and OM have participated in study design and data interpretation and editing. All authors read and approved the final manuscript.

## Pre-publication history

The pre-publication history for this paper can be accessed here:

http://www.biomedcentral.com/1472-6963/11/187/prepub
